# Maternal immune activation upregulates the AU020206-IRFs-STAT1 axis in modulating cytokine production in the brain

**DOI:** 10.7150/thno.96110

**Published:** 2024-09-03

**Authors:** Jing Yang, Wenjun Yu, Runmiao Zhu, Shuangyan Li, Yue Gao, Jinfa Chen, Bin Zhang, Wanshan Wang, Xinping Yang

**Affiliations:** 1 Center for Genetics and Developmental Systems Biology, Division of Nephrology, Nanfang Hospital, Southern Medical University, Guangzhou 510515, China.; 2 Department of Obstetrics & Gynecology, Division of Nephrology, Nanfang Hospital, Southern Medical University, Guangzhou 510515, China.; 3 State Key Laboratory of Organ Failure Research, Division of Nephrology, Nanfang Hospital, Southern Medical University, Guangzhou 510515, China.; 4 Key Laboratory of Mental Health of the Ministry of Education, Guangdong-Hong Kong-Macao Greater Bay Area Center for Brain Science and Brain-Inspired Intelligence and Guangdong Key Laboratory of Psychiatric Disorders, School of Basic Medical Sciences, Southern Medical University, Guangzhou 510515, China; 5 Department of Bioinformatics, School of Basic Medical Sciences, Southern Medical University, Guangzhou 510515, China; 6 Department of Psychiatry, Nanfang Hospital, Southern Medical University, Guangzhou 510515, China; 7 Experimental Animal Center, Southern Medical University, Guangzhou 510515, China.; 8 Lead contact.

**Keywords:** Maternal immune activation, Female offspring, Anxiety, the AU020206-IRFs-STAT1 axis, Cytokine

## Abstract

Maternal immune activation (MIA) is reported to increase the risk of psychiatric disorders in the offspring. However, the underlying mechanism remains unclear.

**Methods**: We constructed a MIA mouse model by intraperitoneal injection of LPS into pregnant mice and evaluated the behaviors and gene expression profiles in the brains of the female and male offspring, respectively.

**Results**: We found that the MIA female offspring exhibited increased anxiety and a large number of differentially expressed genes (DEGs) in the brain, which were enriched with candidate gene sets of psychiatric disorders and immune functions. In contrast, the MIA male offspring exhibited no significant abnormal behaviors and only a small number of DEGs that were not enriched with disease genes and immune functions. Therefore, we further pursued the downstream study on the molecular mechanism underlying the increased anxiety in the female offspring. We identified the lncRNA AU020206-IRFs-STAT1-cytokine axis by integrating lncRNA-protein interaction data and TF-promoter interaction data, and verified the axis *in vitro* and *in vivo*.

**Conclusion**: This study illustrates that MIA upregulates the AU020206-IRFs-STAT1 axis in controlling the brain immunity linked to abnormal behaviors, providing a basis for understanding the role of MIA in psychiatric disorders.

## Introduction

Psychiatric disorders, such as schizophrenia, depression, anxiety disorder and autism spectrum disorder are polygenic diseases that are determined by genetic and environmental factors 1. The injection of poly(I:C) into mice to mimic viral infection can affect brain development during the embryonic stage 2 or early childhood 3, thus increasing the risk of mental illness for the next generation 4. The common feature of these risk factors is the ability to activate the immune system 5. Elevated levels of interleukins are detected in the peripheral blood of patients with psychiatric disorders 6-8, and the immune genes are differentially expressed in the postmortem brain samples of patients with psychiatric disorders 9-11, suggesting an association between immune activation in the blood and the brain of the patients. Researchers have systematically searched for genes and pathways involved in the development of schizophrenia and have identified molecular pathways and regulators linking immune activation to schizophrenia 12.

Epidemiological studies have shown that maternal immune activation (MIA) caused by viral infection, bacterial infection or stress during pregnancy is a risk factor for neurodevelopmental disorders (NDDs) and mood disorders of the offspring, including autism spectrum disorder 13, 14, schizophrenia 15, bipolar disorder 16, depression 17 and anxiety 18. The risk of schizophrenia increases by 7 folds after influenza exposure during the first trimester of pregnancy, but no increased risk of schizophrenia if the exposure is during the second or third trimester 19. How the mother's immune system activation may affect the immune response in the offspring's brain remains unclear.

MIA animal models have been used to explore the development of anxiety 20, depression 21, autism 22 and other central nervous system (CNS) diseases 23. MIA offspring show long-term neuropathological and behavioral changes, including decreased sensorimotor gating, impaired learning and memory, cognitive flexibility defects, increased anxiety, and impaired social ability 23, 24. These findings support that MIA serves as a risk factor for psychiatric disorders.

The behavioral difference between MIA animal models depends on the mouse strain 25, sex 26, the mode and dose of immunogen transmission 27. MIA offspring induced by Poly (I: C) on the 9th day of pregnancy show anxiety behaviors, while in contrast, MIA offspring induced by Poly(I:C) on the 12.5th day of pregnancy in combination with an injection of LPS on the postnatal day 9 show ASD-like behaviors 28. The offspring exposed to Poly(I:C) on the 17th day of pregnancy do not exhibit the aforementioned phenotype 29. The high dose of LPS (120 mg/kg) can directly induce intrauterine fetal mice death 30. Anxiety-like behaviors are found in the offspring with MIA induced by low-dose LPS (100 μg/kg)^31^. Sex differences are observed in MIA models by different researchers using different protocols 32. Numerous studies have been focused on male MIA offspring, and few have been conducted on female MIA offspring. MIA can be transmitted to the fetus and thus affect embryonic brain development 33, 34. However, the molecular mechanism of how MIA leads to psychiatric disorders in the offspring remains unknown. A study on the MIA mouse model has shown that cytokine IL-6 is more easily transferred to the fetus through the placenta during middle pregnancy than in late pregnancy 35, suggesting that some maternal immune factors may mediate the influence of MIA on the developing brain of the offspring. MIA increases the density and activation of microglial cells in different brain regions of the offspring 36-38, the main immune cells in the central nervous system, crucial for brain development and neural protection 39. Diverse evidence suggests that microglia dysfunction is involved in several psychiatric disorders such as schizophrenia 40, autism spectrum disorder 41, bipolar disorder 42, and depression 43.

To identify the molecular changes in the brain of the MIA offspring, we have constructed a MIA mouse model using intraperitoneal injection of LPS (150 μg/kg) into the pregnant mice (GD16) and studied their offspring (P56). Considering the sex difference in mental disorders, we have carried out studies on the female and male offspring respectively, and discovered the AU020206-IRFs-STAT1 axis, which plays a role in upregulating cytokine production in the brain and thus induces abnormal behaviors in the MIA female offspring.

## Methods

### Animal

The C57BL/6J mice were housed under specific pathogen-free (SPF) conditions, 4-5 mice per cage, and maintained on a 12 h light/dark cycle with ad libitum access to food and water. All experimental procedures on animals were approved by the Southern Medical University Experimental Animal Ethics Committee.

### MIA model establishment

On the 16th day of pregnancy, pregnant mice were intraperitoneally injected with 150 μg/kg LPS to induce maternal immune activation. As negative controls, the pregnant mice were injected with an equal volume of saline. Female and male offspring at postnatal day 56 were used for behavioral tests and RNA-seq.

### Behavioral tests

The identities of the offspring were blind to the experimenters during behavioral tests, and the testing order was counterbalanced during the behavioral tests. The mice were placed in the test room at least 2 h before the test.

#### Elevated plus maze test

The elevated plus maze consists of a plus-sign-shaped maze, elevated 50 cm above the floor, with two closed arms (5 cm × 30 cm × 15 cm), two open arms (5 cm × 30 cm × 1 cm) and an intersection (5 cm × 5 cm). During the test, a mouse was placed at the intersection of the arms, facing the open arm. Noldus Video Tracking Software was used to trace the movement of the testing mouse and quantify the time that the mouse spent in the closed or open arms. The arena was cleaned with 75% alcohol after each test.

#### Open field test

The open field test was performed in a rectangular chamber (40 cm × 40 cm × 40 cm) made of gray polyvinyl chloride. Each mouse was placed in the center of the area, and Noldus Video Tracking Software was used to trace the movement of the mouse during the test and quantify the time spent and the distance traveled in the specified area. After each test, the test chamber was cleaned using 75% alcohol.

#### Marble burying test

The marble burying test was performed in a cage containing fresh wood shavings at a depth of 5 cm with a smooth surface. Twenty standard glass marbles (15 mm diameter) were evenly placed on the surface with a 5 × 4 pattern and kept equidistant. The test mouse was then placed in a corner. After 30 min, marbles buried at more than 50% of the shavings were counted.

#### Prepulse inhibition test

The prepulse inhibition tests were performed in automated startle chambers (SM100 M, Kinder Scientific, USA). Each test session consisted of 5 min of acclimatization with only background white noise (70 dB), followed by 42 tests in random order with an average interval of 15 s. Seven types of trials were presented 6 times each in a balanced manner: pulse alone (120 dB, duration: 40 ms), prepulse + pulse (74 dB, 78 dB, 82 dB, 86 dB, 90 dB, duration: 20 ms; intertrial interval (ITI): 100 ms, 20 dB, duration: 40 ms), white noise (70 dB). PPI% was calculated as (1-Prepulse+Pulse/Pulse alone) × 100%.

#### Sucrose preference test

Before the test, the mice were habituated to two identical water bottles for 3 days and were deprived of food and water for 24 h. In the test, the mice were fed two pre-weighed bottles: 1% (w/v) sucrose solution and pure water. After 12 h, the bottles were weighed, and the sucrose preference index was calculated as [sucrose water intake/(sucrose water intake + pure water intake)] × 100%.

#### Forced swimming test

The testing mouse was placed in a transparent plexiglass cylinder (30 cm high, 18 cm diameter) filled with fresh water (25 ± 1 °C) to a depth of 10 cm. After the first 2 min, the immobility time of the mouse was measured by two observers blinded to the treatments for 4 min. The mouse was dried with a towel and the water was changed after each test.

#### Three-chamber social test

The test was performed in three clear plexiglass rectangular chambers (each chamber: 60 cm × 34 cm × 32 cm), which were equipped with partitions that featured doorways permitting the mouse to enter and exit each chamber. Age- and sex-matched mice were utilized as stranger mice and habituated to a wire cage (12 cm high, 11 cm diameter) for 5 days before the beginning of testing. In each test, the testing mouse was first placed in the center chamber with open access to both the left and right chambers, each chamber contained an empty round wire cage, for 10 min of habituation. The wire cage allows nose-to-nose interactions between mice while simultaneously preventing fighting. During the social phase, a stranger mouse was placed in a wire cage, while the opposite cage remained empty. The testing mouse could freely explore the social apparatus for 10 min, interacting with the object (O) or the stranger mouse (S1). The sniffing time was plotted as a social preference index = TS1/(TS1+TO), TS1: time for a testing mouse interacting with a mouse (S1, Stranger1), TO: time for a testing mouse interacting with an empty cage (O, Object). During the second 10 min, a novel mouse was placed in the empty wire cage and the testing mouse was evaluated for its preference for a novel stranger (S2). The sniffing time was plotted as a social preference index = TS2/(TS1+TS2), TS1: time for a testing mouse interacting with a familiar mouse (S1, Stranger 1), TS2: time for a testing mouse interacting with a novel mouse (S2, Stranger 2). The sniffing time (defined as the positioning of the nose of the test mouse within 2.5 cm around a cage) and the chamber time were measured using the Noldus Video Tracking Software.

### RNA preparation

Whole brains from MIA female and male offspring mice and controls on postnatal 8 weeks were used for RNA sequencing. Total RNAs were isolated using the RNeasy Plus Universal Mini Kit (Qiagen, Germany), following the manufacturer's instructions. The quality and yield of the isolated RNAs were assessed using the NanoDrop 2000 (Thermo Fisher Scientific, USA) and Agilent 2100 Bioanalyzer (Agilent Technologies, USA). One microgram of quality-verified RNA was used for library preparation with the NEBNext Ultra^TM^ RNA Library Prep Kit (NEB, USA) and sequenced on Illumina Novaseq (Novogene, China).

### RNA-seq data analysis

After the quality assessment by FastQC (v0.11.8) and the adapters and low-quality reads removed by trimmomatic 44 (v0.39), the sequencing reads were aligned to mouse genome MM10 (UCSC) using STAR 45 (v2.7.1a) under the default parameters. Aligned reads were quantified using HTSeq-counts 46 (v0.11.2). Low-expressed and unexpressed genes with < 3 reads in at least 5 samples were omitted from the analysis (Table S1). Principal component analysis (PCA) was performed by the “prcomp” function in the R-stats package.

Differential expression analysis was performed using the R-edgeR 47 (v2.36.8) and R-DESeq2 48 (v1.24.0) packages. The false discovery rate (FDR) < 0.05 and fold change > 1.3 in both methods were used to define differentially expressed genes (DEGs)(Table S2-3). The heat map was generated by log2(RPKM) of the DEGs using the R-pheatmap package and gene ontology analysis was performed using the R-ClusterProfiler 49 package (v3.12.0).

HOMER 50 was used to search for binding motifs of transcription factors (TFs) in the promoter regions (-2,000 to +1,000 bases of the TSS) of the DEGs. The cutoff p-value for identifying known motifs was 0.05, with the background gene less than 5000. For the De novo motifs, the cutoff match scores were 0.8 and the cutoff p-value was 10e-12. TFs that were not expressed in the brain were removed from the predicted list. TFs were clustered by generating a matrix, with each column representing a TF and each row representing a target gene, according to the TF-target regulation relationship. If the TF regulated the target gene, 1 was assigned; otherwise, it got a default value of 0. The pearson correlation coefficient was calculated between TFs in each column of the matrix, then clustering was carried out according to the correlation coefficient between TFs.

### Rank-rank hypergeometric overlap (RRHO)

Rank-rank hypergeometric overlap (RRHO) was used to evaluate the overlap of differential expression lists without cutoffs. RRHO maps were produced by calculating the normal approximation of the difference in the log odds ratio and standard error of overlap between female and male offspring. The Zscore was converted to a p-value and corrected for multiple comparisons across the pixels. The -log10(p-value) multiplied by the sign of the fold change in expressed genes was used to calculate the consistency of gene expression among different groups by RRHO package 51 (v1.24.0), mapping the degree of the statistical significance of overlaps between two differential transcriptomes (two ranked gene lists on the X-axis or Y-axis).

### Psychiatric disorder gene collection

Autism candidate genes were collected from AutDB 52, SFARI .0 53, and AutismKB 2.0 54 databases. Schizophrenia candidate genes were collected from 7 different methods in 87 studies 12. Anxiety candidate genes were collected from 31 studies by using “anxiety” as the keyword to search the PubMed database. Depression candidate genes were collected by searching PubMed with “depression” as the keyword. Immune genes were collected from the InnateDB database 55, including the following parts: InnateDB Innate Immune Genes, ImmPort (Immunology Database and Analysis Portal), IRIS (Immunogenetic Related Information Source) and Immunome Database (Table S4-5).

### lncRNA-TF-target network construction

The lncRNA-protein interactions were collected from the RNAInter v4.0 database 56. Only lncRNA-protein interactions with the “Mus musculus” organism and experiment evidence were kept. Differentially expressed lncRNAs and IRFs-STAT1 interactions were mapped to the lncRNA-protein interaction network to retrieve the DE lncRNA-IRFs-STAT1 network.

### BV2 cell culture

The BV2 microglial cell line was obtained from American Type Culture Collection (USA). The cells were cultured in high-glucose DMEM (GIBCO, USA) containing 10% fetal bovine serum (GIBCO, USA) and incubated at 37 ℃ in a suitable atmosphere containing 95% air and 5% CO_2_.

### Primary microglial cell culture

Primary mouse microglial cells were isolated from the cerebral cortices of 1-day-old neonatal MIA offspring and control offspring. The meninges were removed and the cortical tissues were washed in D-Hanks (Corning, USA) and digested with 0.25% trypsin-EDTA (Corning, USA) for 20 min at 37 ℃, followed by grinding in DMEM/F12 (Corning, USA) with 10% fetal bovine serum (Gibco, USA). The tissues were passed through a 70-um nylon mesh cell strainer (Solarbio, China) and cultured in DMEM/F12 (Corning, USA), supplemented with 10% fetal bovine serum (Gibco, USA). After 1 h, the non-adherent cell suspension was isolated and cultured in DMEM/F12 (Corning, USA) with 10% fetal bovine serum (Gibco, USA) for 9 days. The microglial cells were isolated from the mixed glial cultures by shaking.

### Reverse-transcribed PCR and quantitative real-time PCR

Total RNAs were isolated and reverse-transcribed using the PrimeScript^TM^ RT Reagent Kit (Takara, Japan). Quantitative real-time PCR was performed using SYBR Premix^TM^ Ex Taq^TM^ Kit (Takara, Japan) on the Applied Biosystems 7500 FAST Real-Time PCR system (Thermo Fisher Scientific, USA). The primers are listed in Table S8. The relative expression levels of the genes were recorded using the 2 (-delta delta CT) method, with β-actin as the internal control.

### Chromatin immunoprecipitation assay (ChIP)

The coding sequences of IRF1 or IRF2 were cloned into the pcDNA3.1-Flag vector (GeneChem, China). The plasmids were transfected into BV2 cells using Lipofectamine 3000 (Invitrogen, USA). ChIP assays were performed using the SimpleChIP Chromatin IP Kit (Cell Signaling Technology, USA), following the manufacturer's instructions. Anti-FlAG antibodies were obtained from Cell Signaling Technology (USA). The qRT-PCR was conducted to assess the enrichment of immunoprecipitated DNA from the ChIP experiment.

### Lentiviral vector infection

To overexpress AU020206, IRF1 and IRF2, BV2 cells were infected with lentiviral particles carrying AU020206, IRF1, IRF2 (GeneChem, China), respectively. To knockdown AU020206, IRF1 and IRF2, BV2 cells were infected with lentiviral particles carrying AU020206 shRNAs, IRF1 shRNAs, IRF2 shRNAs (Fenghbio, China), respectively.

### Nuclear/cytoplasm fractionation

Cytosolic and nuclear fractions of BV2 cells were prepared using NE-PER™ Kit (Thermo Fisher Scientific, USA) following the manufacturer's instructions. Briefly, cells were collected after trypsinization, washed 3 times in PBS, and then fractioned into cytoplasmic and nuclear fractions using cell fractionation buffer. The nuclear fraction was washed and disrupted in cell disruption buffer. The RNAs from the cytoplasmic and nuclear components were extracted separately and reverse-transcribed into cDNAs. The expression levels of AU020206 were evaluated by qRT-PCR, with GAPDH and U6 as the cytoplasmic and nuclear controls, respectively.

### Chromatin isolation by RNA purification (ChIRP)

The interactions between AU020206 and IRF1 or IRF2 were detected using the CHIRP Kit (Saichengbio, China) according to the manufacturer's instructions. BV2 cells were treated with formaldehyde for cross-linking. Stop solution (with 125 mM glycine) was added to the cell lysates to stop cross-linking. Cells were collected after trypsinization and resuspended in cell lysis buffer with RNase inhibitors and proteinase inhibitors. The cell suspension was sonicated to fragment the chromosomes into 100-500 bp. The biotinylated lncRNA probes (100 pmol) or biotinylated lacZ oligos (Saichengbio, China) were incubated with the cell lysates for 4 h. Streptavidin magnetic beads were washed with lysis buffer, added to the cell lysates, and incubated at 37 ℃ for 30 min. The supernatants were removed, and the beads were washed with lysis buffer 5 times. The proteins were extracted from the beads for the western blot.

### Western blot

The protein expression levels of IRF1, IRF2 and STAT1 in the BV2 cells were measured by western blot. The cells were washed with PBS and lysed in lysis buffer containing protease and phosphorylase inhibitors at 4 ℃. The protein concentration was determined using the BCA Protein Assay Kit (KeyGEN, China). Total proteins from BV2 cells were denatured, separated by sodium dodecyl sulfate-polyacrylamide gel electrophoresis containing 8%-10% acrylamide, and subsequently transferred to PVDF membranes. PVDF membranes were blocked with 5% skimmed milk powder and subsequently incubated with antibodies against IRF1 (Cell Signaling Technology, USA), IRF2 (Cell Signaling Technology, USA), STAT1 (Cell Signaling Technology, USA), or GAPDH (Cell Signaling Technology, USA) respectively, overnight at 4 °C. Antibodies were diluted according to the manufacturer's instructions. The membranes were washed with TBST, incubated with the secondary antibody for 2h, and then the protein bands were visualized using a BeyoECL Plus Kit (Beyotime, China).

### RNA immunoprecipitation (RIP)

RNA immunoprecipitation experiments were performed using the BeyoRIP™ RIP Assay Kit (Beyotime, China). BV2 cells were collected via centrifugation, lysed in RIP lysis buffer, and immunoprecipitated. The cell lysates were mixed with protein A/G agarose-antibody complexes and incubated at 4 ℃ for 4 h. The complex was washed in the elution buffer and immunoprecipitated RNAs were examined using qRT-PCR.

### Luciferase reporter assay

The STAT1 promoter sequence was cloned into a PGL3-Luci vector. Promoter activity was evaluated using the Luciferase Assay Kit (Yijinbio, China) with the empty plasmid vector pRL-TK as a control. PGL3-Luci-pSTAT1 and pRL-TK plasmids were transfected into cell lines with stable overexpression or knockdown of AU020206. After 48 h, the culture medium was discarded, 1 × Luc LyII Buffer was used to lyse cells and FLuc solution (100 μl) was added to the culture plates. Fluorescence levels were determined using the Varioskan^TM^ LUX multimode microplate reader (Thermo Fisher Scientific, USA).

### Lentiviral expression constructs and small interfering RNA (siRNA) transfection

To produce viral particles, lncRNA AU020206 was cloned into the lentiviral vector pGC-FU (Genechem, China) and transfected into HEK293T cells, following the manufacturer's instructions. After viral particles were transfected into BV2 cells, stable cell lines of overexpression and knockdown AU020206 were selected using puromycin (Gibco, USA). The siRNAs of STAT1 (GeneChem, China) were transfected into BV2 cells using the Lipofectamine 3000 (Invitrogen, USA), according to the manufacturer's instructions. After 48h transfection, cells were harvested for further experiments. The sequences of siRNAs are listed in Table S8.

### ELISA assay

The levels of TNF-α, IL-6 and IL-1β were measured using ELISA kit (Renjiebio, China) following the manufacturer's instructions. The supernatant from the BV2 cell culture was collected and added to the test wells of an ELISA plate, and 50 μl of each standard sample at different concentrations was added to the standard wells. Horseradish peroxidase-labeled antibodies (100 μL) were incubated with the samples in a 37 °C water bath for 60 min. The liquid was discarded and the wells were dried with absorbent paper. The wells were washed five times with the washing buffer 5 times. A volume of 50 μl substrate was added to each well and incubated at 37 ℃ in the dark for 15 min. Termination solution(50 μl) was added to each well. The OD value of each well was measured using a Varioskan^TM^ LUX multimode microplate reader (Thermo Fisher Scientific, USA) at a wavelength of 450 nm.

### Overexpression of lncRNA in the mouse brain

The lncRNA AU020206 was cloned into an adeno-associated virus (AAV) vector with the IBA1 promoter and introduced into HEK293T AAV packing cells to produce AAV viral particles (Shengbobio, China). The titration of the purified virus was 1.4 × 10^12^ µg/ml. The AAVs (1μl per mouse) were stereotaxically injected into the right prefrontal lobe of 8-week-old female mice at stereotactic coordinates (bregma: ML: 0.4 mm, AP: 1.7 mm, DV: -2.3 mm). Three weeks after AAV injection, the prefrontal tissues of the mice were subjected to qRT-PCR to examine the expression levels of IRF1, IRF2, STAT1, and cytokines. Behavioral tests were performed to assess anxiety and depression.

### Fluorescent *in situ* hybridization and immunofluorescence

Three weeks after AAV injection, prefrontal cortex tissues were isolated and post-fixed in 4% PFA overnight, 8-μm-thick paraffin sections were deparaffinized and rehydrated using xylene and alcohol, washed with PBS. The probe of AU020206 was obtained from SaichengBio (China). The localization and distribution of AU020206 were detected using the Fluorescence *In Situ* Hybridization Kit (SaichengBio, China) following the manufacturer's instructions. The slides were microwaved in Tris-EDTA (PH 9.0) for antigen retrieval. Slides were blocked with 1% BSA for 1 h. The slides were then incubated with IBA1 antibody (Cell Signaling Technology, USA) overnight at 4 ℃, followed by incubation with donkey anti-rabbit-647 secondary antibody (Abcam, USA) at room temperature for 1 h. The slides were washed with PBS and incubated with DAPI (Sigma, USA). Quantification of AU020206 and IBA1 on the slides was performed by the confocal microscope (Zeiss, Germany).

### Statistical analysis

Gene enrichment analysis was performed using the single-sided fisher test. Error bars represent the standard error of the fraction, estimated using a bootstrapping method with 1000 resamplings. No outliers in behavioral tests with more than 2 standard deviations from the group mean were detected. The homoscedasticity and normality of the data distributions were determined using GraphPad Prism 9 before assigning specific statistical tests. Where normality and equal variance between sample groups were achieved, a two-tailed unpaired student's t-test was used. Behavioral data were expressed as means ± SEM values. The molecular experiment data were represented as mean ± standard deviation of three biological replicates (unless specified otherwise in figure legends). The significance was determined by the two-tailed unpaired student's t-test and labeled in the figures as follows: not significant (ns); * p < 0.05; ** p < 0.01; *** p < 0.001; **** p < 0.0001.

## Results

### The MIA female offspring exhibit increased anxiety, but the male does not

**Figure 1:**
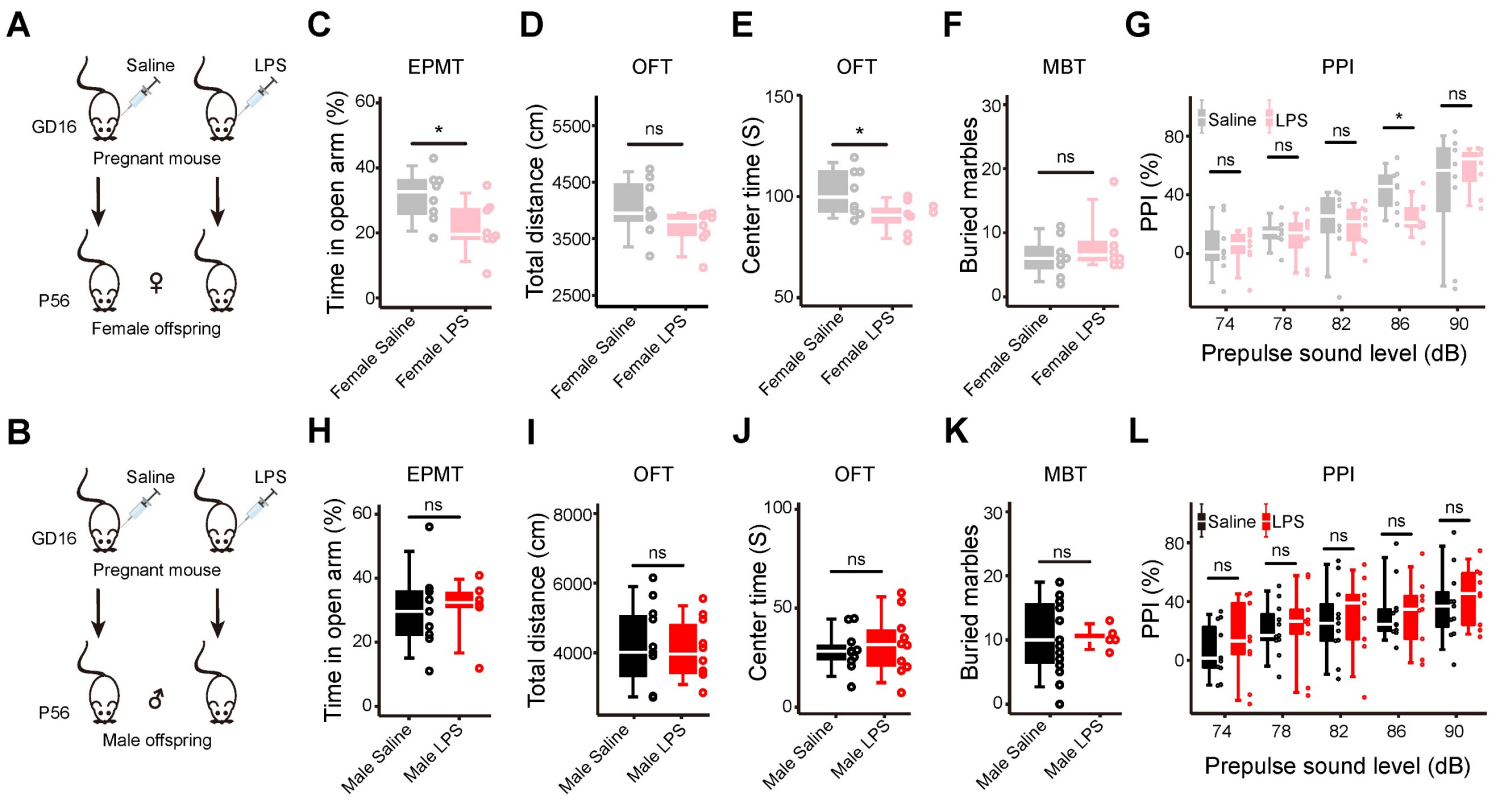
** Maternal immune activation increases anxiety-like behaviors in female offspring, but not in the male offspring. (A)** The schematic of the MIA model in the female offspring. LPS: lipopolysaccharide; GD16: gestational day 16; P56: postnatal day 56. **(B)** The schematic of the MIA model in the male offspring. **(C)** The elevated plus maze test (EPMT) of the female offspring. The time spent in the open arms by MIA female offspring is compared with that of the controls (p = 0.03, n = 8 for each group). **(D)** The open field test (OFT) of the female offspring. The total distance traveled by the MIA female offspring is compared with that of the controls (p = 0.12, n = 8 for each group). **(E)** The open field test (OFT) of the female offspring. The time spent in the center zone by the MIA female offspring is compared with that of the controls (p = 0.03, n = 8 for each group). **(F)** The marble burying test (MBT) of the female offspring. The number of marbles buried by the MIA female offspring is compared with that of the controls (p = 0.34, n = 8 for each group). **(G)** The prepulse inhibition test (PPI) of the female offspring. The percentage of prepulse inhibition of the MIA female offspring is compared with those of the controls under different prepulse strengths (p = 0.93 for 74 dB; p = 0.59 for 78 dB; p = 0.85 for 82 dB; p = 0.02 for 86 dB, p = 0.37 for 90 dB; n=8 for each group). **(H)** The elevated plus maze test of the male offspring. The time spent in the open arms by the MIA male offspring is compared with that of the controls (p = 0.92, n = 9 for the saline group, n = 6 for the LPS group). **(I)** The open field test of the male offspring. The total distance traveled by the MIA male offspring is compared with that of the controls (p = 0.81, n = 11 for the saline group, n = 10 for the LPS group). **(J)** The open field test of the male offspring. The time spent in the center zone by the MIA male offspring is compared with that of the controls (p = 0.57, n = 11 for the saline group, n = 10 for the LPS group). **(K)** The marble burying test of the male offspring. The number of marbles buried by the MIA male offspring is compared with that of the controls (p = 0.83, n = 19 for the saline group, n = 6 for the LPS group). **(L)** The prepulse inhibition test of the male offspring. The percentage of prepulse inhibition of the MIA male offspring is compared with those of the controls under different prepulse strengths (p = 0.52 for 74 dB; p = 0.90 for 78 dB; p = 0.71 for 82 dB; p = 0.96 for 86 dB; p = 0.53 for 90 dB; n = 11 for the saline group, n = 9 for the LPS group). Data are presented as boxplots showing the median, the quantiles, and the 5th-95th percentile whiskers. The data values are shown as dots along the boxes, student's t-test; ns: not significant; * p < 0.05.

To systematically study the behaviors and molecular events in the brains of MIA offspring, we generated a MIA mouse model by intraperitoneal injection of LPS (150 μg/kg) into the pregnant mice on gestational day 16 (GD16). Considering the sex difference in psychiatric disorders, we carried out a repertoire of behavioral tests on the female (**Figure 1A**) and male offspring (**Figure 1B**) at postnatal 8 weeks, respectively. In the elevated plus maze test (EPMT), the female offspring spent significantly less time in the open arms (n = 8 for each group, p = 0.03, **Figure 1C**). Less time spent in the open arms indicates increased anxiety 57. In the open field test (OFT), the female offspring showed no difference in total distance within 30 min compared with the control offspring (n = 8 for each group, p = 0.12,** Figure 1D**), suggesting normal locomotion ability 58. The MIA female offspring spent significantly less time in the center zone during the first 5 min (n = 8 for each group, p = 0.03,** Figure 1E**), which also suggests increased anxiety 58. In the marble burying test (MBT), the female offspring buried more marbles, but without statistical significance (n = 8 for each group, p = 0.34, **Figure 1F**). Increased marble-burying behavior can be interpreted as anxiety 59. In the prepulse inhibition test (PPI), the MIA female offspring showed lower PPI% at 86 dB (n = 8 for each group, p = 0.024), and a trend of decreasing PPI% with the increasing prepulse strength, compared with the control offspring (n = 8 for each group. p = 0.93 for 74 dB, p = 0.59 for 78 dB, p = 0.85 for 82 dB, p = 0.37 for 90 dB. **Figure 1G**). The prepulse inhibition test is used to evaluate the sensor-gating ability of the brain 60. Impaired prepulse inhibition is often observed in patients with psychiatric disorders 61. In the sucrose preference test (SPT), they showed no sucrose preference (n = 8 for each group, p = 0.24, **Figure S1A**), suggesting that the MIA female offspring have no anhedonia 62. Neither did they show a change in the duration of immobility in the forced swimming test (FST) (n = 8 for each group, p = 0.73, **Figure S1B**), suggesting that the MIA female offspring have no helplessness behavior. Both results of SPT and FST suggest no depression behaviors in the MIA female offspring. We also carried out most of the above behavioral tests on the male offspring and found that the MIA male offspring showed no abnormal behaviors, compared with the control male offspring (**Figure 1H-L, S1C-D**). These results show that the MIA female offspring have anxiety behaviors, but the male does not.

### The genes involved in immune functions and psychiatric disorders are dysregulated in the MIA female offspring

**Figure 2:**
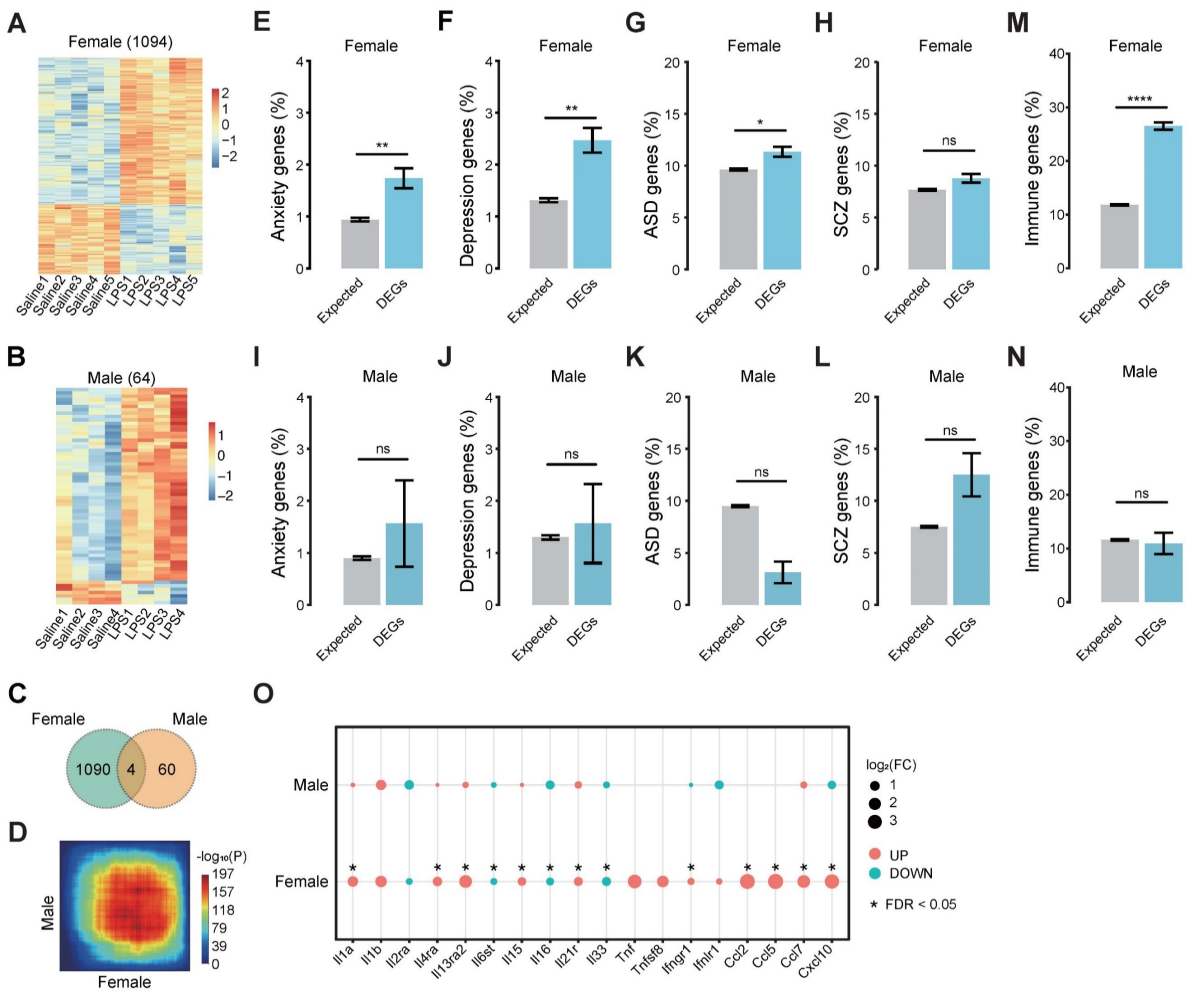
** The transcriptomic dysregulation in the brain of the MIA offspring. (A)** The heat map of the 1,094 DEGs of the female offspring. **(B)** The heat map of the 64 DEGs of the male offspring. **(C)** The Venn diagram of the DEGs of MIA female and male offspring. **(D)** The threshold-free comparisons of global differential expression by rank-rank hypergeometric overlap (RRHO)^51^ between female and male offspring. Pixels represent the overlap between the transcriptomes of each comparison (LPS versus Saline), with the significance of overlap (-log10(p-value) of a hypergeometric test) color-coded. Genes along each axis were sorted from most significantly up-regulated to most significantly down-regulated. **(E-H)** The enrichment of candidate gene sets of anxiety **(E)**, depression **(F)**, ASD **(G)**, SCZ **(H)** in the DEGs of MIA female offspring. **(I-L)** The enrichment of candidate gene sets of anxiety **(I)**, depression **(J)**, ASD **(K)**, SCZ **(L)** in the DEGs of MIA male offspring. **(M)** The enrichment of immune genes in the DEGs of the MIA female offspring. **(N)** The enrichment of immune genes in the DEGs of the MIA male offspring. **(O)** The differential expression levels of cytokines in MIA male and female offspring. Up-regulated cytokines are red nodes and down-regulated ones are blue nodes. The size of nodes represents the log2 (FC) of the cytokines. The asterisks indicate the FDR < 0.05. Error bars represent the standard error of the fraction, estimated using a bootstrapping method with 1000 resamplings. One-tailed fisher's exact test, * p < 0.05, ** p < 0.01, *** p < 0.001, **** p < 0.0001.

To further explore the underlying molecular mechanisms on how MIA may cause increased anxiety in the female offspring, we carried out RNA-seq on the whole brain of the female and male offspring on postnatal 8 weeks. The raw RNA-seq reads were subjected to quality control steps including adapter cleavage and low-quality reads removal before downstream analysis (Table S1). We performed differential expression analysis using edgeR and DESeq2 methods and obtained well-consistent DEGs using a cutoff of FDR < 0.05 and FC > 1.3 in both methods. We took the intersection of DEGs identified by both methods for downstream analysis: 1,094 DEGs for female offspring (**Figure 2A,** Table S2) and 64 DEGs for male offspring (**Figure 2B,** Table S3). We compared the MIA-induced differential expression in female versus male offspring mice and found that the male mice shared 0.37% (4/1,094) DEGs in the female mice (**Figure 2C**). Then we used rank-rank hypergeometric overlap (RRHO) analysis in a threshold-free manner to compare the gene expression changes between female and male MIA offspring. The female and male offspring showed different expression change patterns (**Figure 2D**). These results show that the MIA induces a much stronger effect on the gene expression in the brain of the female offspring than the male offspring.

To evaluate the MIA-induced gene expression changes that might have impacted the brain functions associated with psychiatric disorders, we further performed the enrichment analysis of the candidate gene sets of several psychiatric disorders (Table S4) in the DEGs of both sexes MIA offspring respectively. The DEGs of the MIA female offspring mice were enriched with the candidate gene sets of anxiety (**Figure 2E**, 1.7%, p = 7.4e-3), depression (**Figure 2F**, 2.5%, p = 1.3e-3), autism spectrum disorder (ASD) (**Figure 2G**, 11.3%, p = 0.03), and exhibited a trend of enrichment with the schizophrenia (SCZ) candidate gene (**Figure 2H**, 8.8%, p = 0.09). The DEGs of the MIA male offspring only showed a trend of enrichment with these gene sets (**Figure 2I-L**). We further performed Gene Ontology enrichment analysis on the DEGs. The DEGs of MIA female offspring were enriched in biological processes mostly associated with immunity, such as response to virus, cytokine production, and immune effector process, and also include terms related to neuron death (**Figure S2A**), while the DEGs of the MIA male offspring showed enrichment in some energy metabolic pathways (**Figure S2B**). Consistent with the biological process enrichment, the DEGs of the MIA female offspring were enriched with immune genes (**Figure 2M**, 26.5%, p = 5.3e-43, Table S5), while the DEGs of the male offspring were not (**Figure 2N**). Many cytokines were differentally expressed in the MIA female offspring, but not in the MIA male offspring (**Figure 2O**). These results suggest that MIA may induce significant dysregulation of immunity in the brain of female offspring, leading to observable behavioral changes, while it induces much less effect on male offspring.

### The IRFs-STAT1 regulatory network controls the gene dysregulation of the brain in the MIA female offspring

**Figure 3:**
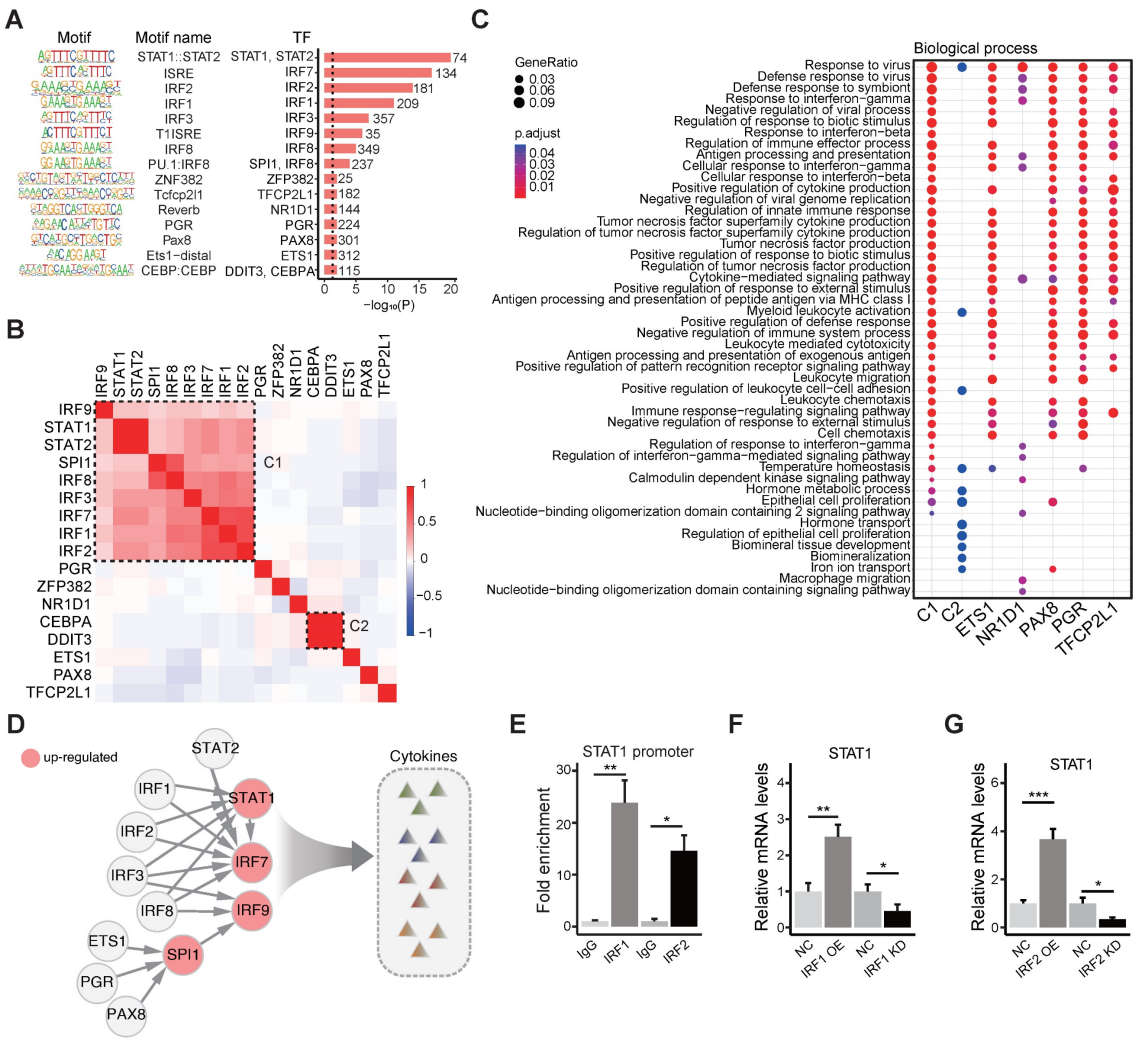
** The IRFs-STAT1 regulatory network controls the expression of genes in the brain of MIA female offspring. (A)** The binding motifs identified in the promoters of DEGs of MIA female offspring using HOMER 50. The motif sequences are shown on the left. The bar plot shows the p-value and target DEG number of each TF. **(B)** The heatmap of TF clustering based on shared targets. The scaled color shows the comparison pairs of TFs with correlation coefficients. **(C)** The comparison of the enriched biological processes of the TF targets. The sizes of the dots represent the gene ratio and the colors represent the adjusted p-value. **(D)** The diagram of the IRFs-STAT1 regulatory network. The arrows show the regulation relationships between the TFs in controlling the cytokine expression. Up-regulated TFs are highlighted in red. **(E)** The bindings of IRF1 and IRF2 to the promoter of STAT1 using ChIP assays followed by qRT-PCR in BV2 cells, respectively. **(F)** The relative expression levels of STAT1 in BV2 cells with IRF1 overexpression and knockdown, detected by qRT-PCR, respectively. **(G)** The relative expression levels of STAT1 in BV2 cells with IRF2 overexpression and knockdown, detected by qRT-PCR, respectively. Student's t-test; Data are shown as the mean ± SD of three independent experiments; * p < 0.05, ** p < 0.01, *** p < 0.001.

The transcriptional patterns are determined by factors regulating gene transcription globally and locally, including transcription factors (TFs), epigenetic factors, and noncoding RNAs. We identified 18 TFs that had enriched binding motifs in the promoters of the MIA female offspring DEGs using HOMER 50. Of these 18 TFs, 17 show expression in the brain (**Figure 3A**, Table S6).

We speculated that these TFs might cooperate in regulating the DEGs, and therefore, we clustered the TFs according to the correlation coefficient between the TFs based on shared target genes (**Figure 3B**, Table S7). Most TFs were clustered into one cluster (C1), which includes IRF and STAT family members: IRF9, STAT1, STAT2, SPI1, IRF8, IRF3, IRF7, IRF1 and IRF2. The second cluster (C2) includes C/EBP family members: CEBPA and DDIT3. The other 6 TFs were isolated outside these two clusters. TFs in the C1 cluster have their binding motif in about 45.4% (497/1,094) DEGs of the MIA female offspring, suggesting that IRF and STAT family may be the major TFs involved in the differential expression in the MIA female offspring brain (**Figure S3A**).

We performed enrichment analysis on the Gene Ontology biological processes for the target genes of C1, C2, and other isolated TFs. We found that the targets of C1 were enriched in immune functions such as response to virus, immune effector process, cytokine production, etc., and the isolated TFs, ETS1, PAX8, PGR, and TFCP2L1 were enriched with similar functions of C1. The C2 cluster and NR1D1 showed less enrichment in immune functions (**Figure 3C**).

To explore the regulatory relationship between the TFs, we searched these TF-binding motifs in the promoter of each TF and obtained a tight regulatory network of these TFs (**Figure 3D**). Some inter-regulations exist in IRF family members, and most IRF family members target STAT1. To validate the regulation of IRFs on STAT1, we carried out chromatin immunoprecipitation (ChIP) using the anti-FLAG antibody, followed by qRT-PCR, which verified the binding of IRF1 and IRF2 to the promoter of STAT1 (**Figure 3E**). We further carried out overexpression or knockdown of IRF1 and IRF2 in BV2 cells, respectively, and found that the overexpression of IRF1 or IRF2 upregulated STAT1, while the knockdown of IRF1 or IRF2 downregulated STAT1 (**Figure 3F-G**). STAT1 is known to control the production of cytokines 63, which is consistent with the enriched biological processes (**Figure 3C**). These results suggest that IRFs may regulate the expression of STAT1, leading to cytokine production in immune activation. However, most IRFs (IRF1/2/3/8) that regulate STAT1 are only slightly up-regulated (**Figure S3B**), which can partially explain the increased expression level of STAT1. Therefore, besides these transcription factors, other factors would regulate the transcription of STAT1, including some co-factors and lncRNAs.

### AU020206 binds IRF1/IRF2 to regulate transcription of STAT1 in controlling cytokine production

**Figure 4:**
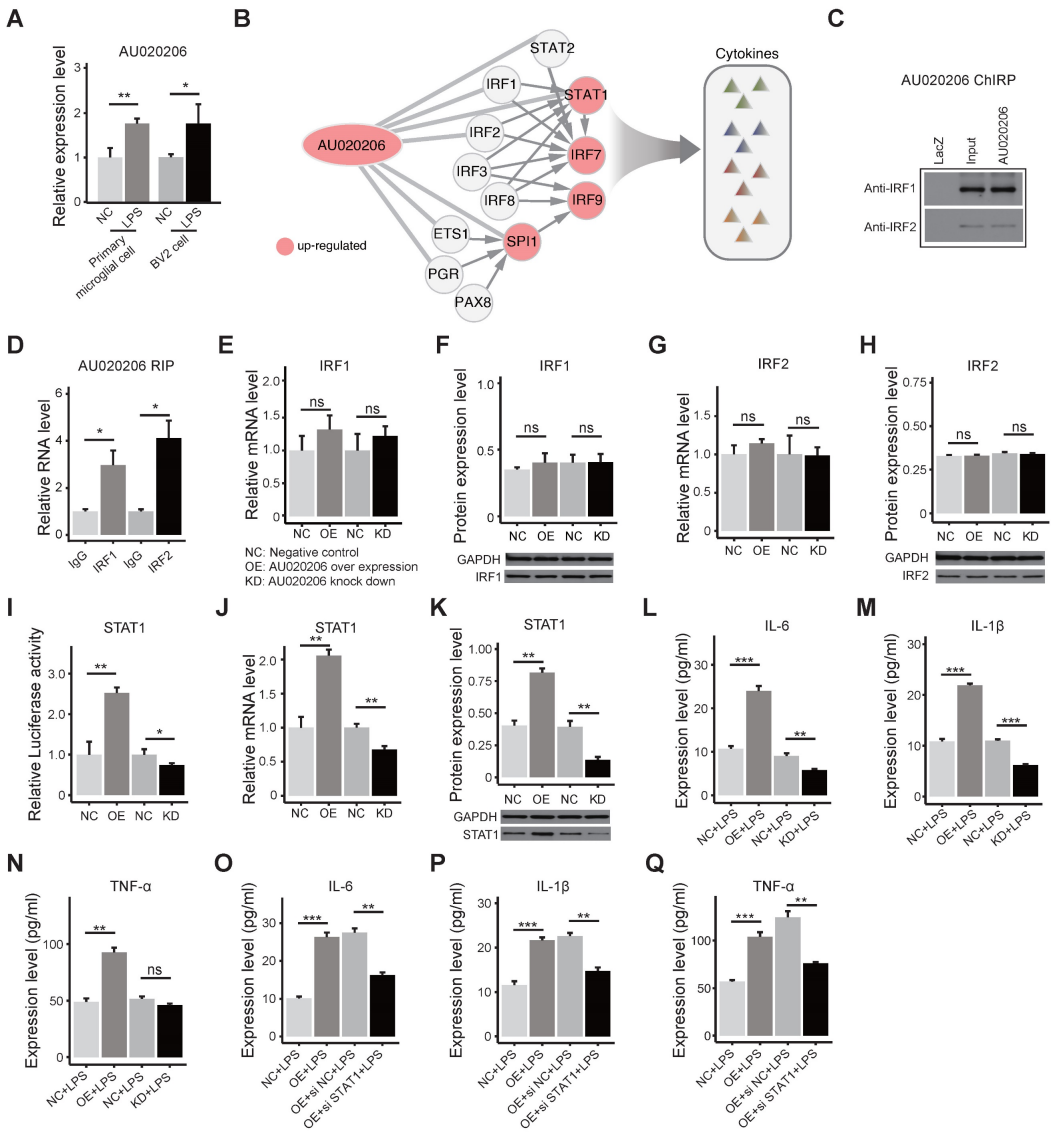
** AU020206 interacts with IRF1/IRF2 to regulate the transcription of STAT1 in controlling cytokine production. (A)** The expression of AU020206 in the primary microglial cells of LPS-induced MIA offspring on postnatal one-day and BV2 cells treated with LPS, respectively. **(B)** The AU020206-IRFs-STAT1-cytokine regulatory network. Red nodes represent up-regulated genes. The arrow edges represent the regulation relationship between TFs. The light gray lines represent AU020206-TFs interactions. **(C)** The verification of the interactions between AU020206 and IRF1/IRF2 using ChIRP followed by western blot, respectively. **(D)** The verification of the interaction of AU020206 and IRF1/IRF2 using RIP assay followed by qRT-PCR, respectively. **(E-H)** The relative mRNA and protein expression levels of IRF1 **(E, F)** and IRF2 **(G, H)** in BV2 cells with AU020206 overexpression or knockdown, detected by qRT-PCR and western blot, respectively. **(I)** The transcriptional activity of STAT1 promoter in BV2 cells with AU020206 overexpression or knockdown, detected by luciferase reporter assay, respectively. **(J-K)** The relative mRNA **(J)** and protein **(K)** expression levels of STAT1 in BV2 cells with AU020206 overexpression or knockdown, detected by qRT-PCR and western blot, respectively. **(L-N)** The expression levels of cytokines IL-6 **(L)**, IL-1β **(M)** and TNF-α **(N)** in BV2 cells (treated with LPS) with AU020206 overexpression or knockdown, detected by ELISA, respectively. **(O-Q)** The expression levels of cytokines IL-6 **(O)**, IL-1β **(P)** and TNF-α **(Q)** in BV2 cells (treated with LPS) with AU020206 overexpression and STAT1 knockdown, detected by ELISA, respectively. Student's t-test; Data are shown as the mean ± SD of three independent experiments; ns: not significant, * p < 0.05, ** p < 0.01, *** p < 0.001.

Considering that other factors, such as lncRNAs, might also regulate the activity of these TFs in the regulatory network, we analyzed the differential expression of lncRNAs, and we found 78 differentially expressed lncRNAs in the brains of MIA female offspring. By mapping the IRFs-STAT1 network and DE lncRNAs into the published lncRNA-protein interaction network RNAInter v4.0 56, we identified 56 DE lncRNAs that interact with the TFs in the IRFs-STAT1 network (**Figure S4A**). These 56 DE lncRNAs are considered candidates for regulating the activity of these transcription factors. To pick a candidate for functional validation, the DE lncRNAs were ranked by the extent of differential expression and the number of interacting TFs (**Figure S4B**). The DE lncRNAs AU020206, 0610040B10Rik, and 6530402F18Rik interact with 7 TFs in the regulatory network, and the fold change of AU020206 is the biggest (2.03) among the three lncRNAs (**Figure S4B**). We verified that AU020206 was up-regulated in the primary microglial cells from the MIA offspring on postnatal day 1 (**Figure 4A**), and we also detected its upregulation in the BV2 cells (a microglial cell line) treated with LPS (**Figure 4A**). Therefore, we further constructed the “AU020206-IRFs-STAT1-target regulatory network” by integrating the “AU020206-TF interactions” and “IRFs-STAT1 regulatory network”, which indicate that AU020206 may interact with IRFs to regulate the expression of STAT1 in controlling cytokine production (**Figure 4B**). To prove that AU020206 regulates STAT1 transcription by interacting with IRF1/IRF2 in BV2 cells, we first detected the nuclear localization of AU020206 by subcellular fractionation (**Figure S4C**). Then we validated the physical interactions between IRF1/IRF2 and AU020206, by pulling down AU020206 followed by western blot against the IRF1/IRF2 (**Figure 4C**) and by IRF1/IRF2 immunoprecipitation followed by qRT-PCR on AU020206 (**Figure 4D**). The overexpression or knockdown of AU020206 had no significant effect on the expression of IRF1 and IRF2 in BV2 cells at mRNA and protein levels (**Figure 4E-H**). We further carried out a luciferase assay to check the promoter activity of STAT1 in BV2 cells with overexpression or knockdown of AU020206. We found that AU020206 overexpression promoted the transcription activity of the STAT1 promoter, whereas AU020206 knockdown inhibited the transcription activity (**Figure 4I**), and AU020206 overexpression promoted the expression of STAT1 at both mRNA and protein levels, while knockdown AU020206 reduced its expression (**Figure 4J-K**).

Based on the cytokine production regulation of the AU020206-IRFs-STAT1 regulatory network (**Figure 4B**), we performed overexpression and knockdown of AU020206 in BV2 cells treated with LPS to test its effect on the expression of the inflammatory cytokines. Overexpression of AU020206 promoted the release of inflammatory cytokines (IL-6, IL-1β and TNF-α) in BV2 cells treated with LPS, and knockdown of AU020206 inhibited the release of IL-6 and IL-1β (**Figure 4L-N**). We further knocked down STAT1 together with AU020206 overexpression in BV2 cells treated with LPS and found that the knockdown of STAT1 inhibited the release of the cytokines induced by AU020206 (**Figure 4O-Q**). These results demonstrate that AU020206 binds IRF1/IRF2 to promote the transcription of STAT1, which further promotes the transcription of cytokines, illustrating the AU020206-IRFs-STAT1-cytokine axis.

### Overexpression of AU020206 in the prefrontal cortex upregulates STAT1 and cytokines and leads to enhanced anxiety behaviors in female mice

**Figure 5:**
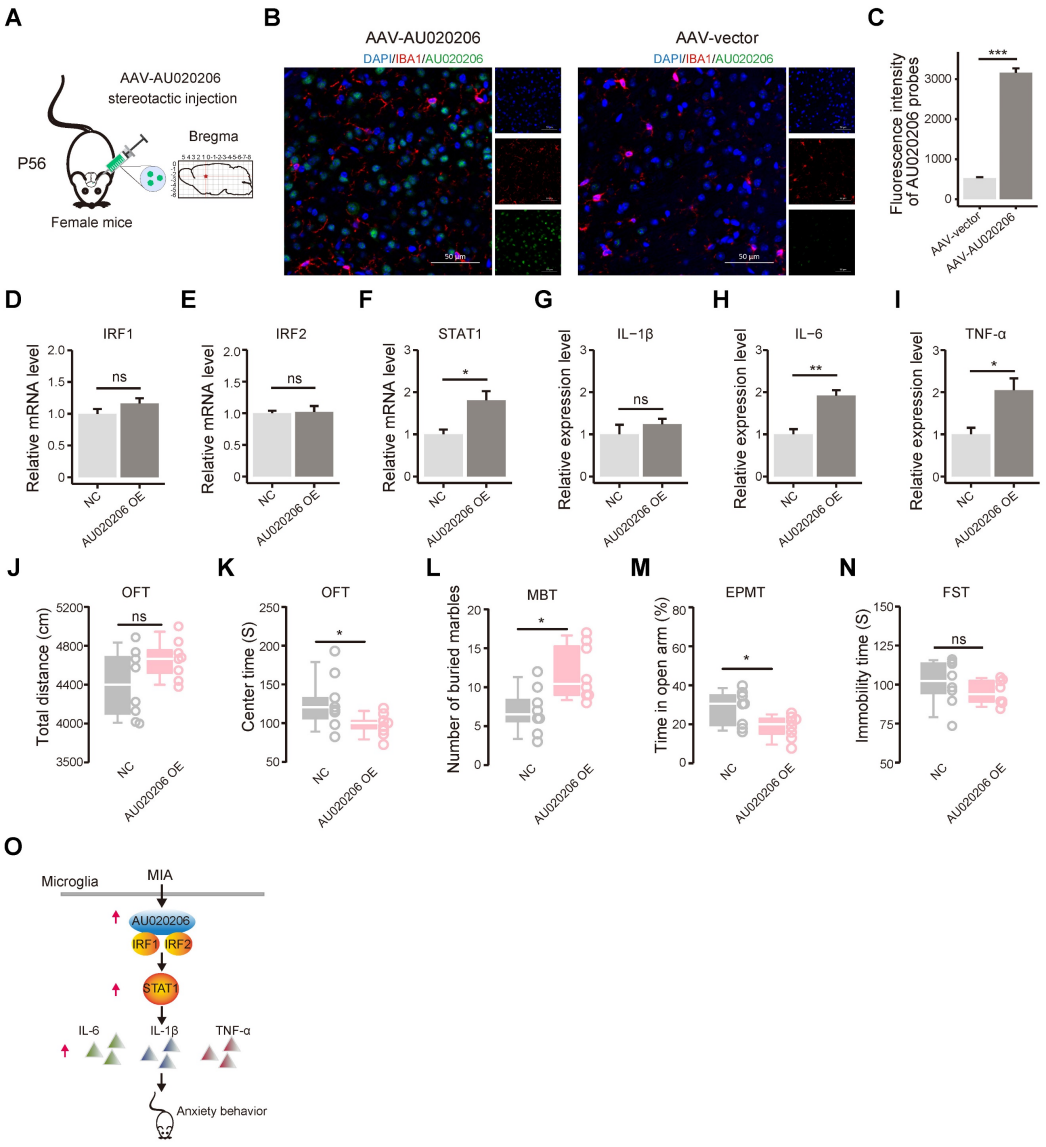
** Overexpression of AU020206 promotes STAT1-mediated cytokine production and induces anxiety behaviors in female mice. (A)** A schematic for the prefrontal stereotactic injection of AAV carrying AU020206 into female mice. Position: AP: 1.7 mm. DV: -2.3 mm. ML: 0.4 mm (right). P56: postnatal day 56. **(B)** Representative immunofluorescence (IF) images for IBA1 and fluorescence *in situ* hybridization (FISH) images for AU020206 of the prefrontal cortex. DAPI: nuclear staining; IBA1: microglia marker; Scale bars: 50 μm. **(C)** The relative fluorescence intensity of AU020206 probes detected by FISH, using the immunofluorescence intensity of DAPI as the internal control. **(D-F)** The relative expression levels of IRF1 **(D)**, IRF2 **(E)** and STAT1 **(F)** in the prefrontal cortex, detected by qRT-PCR, respectively. **(G-I)** The relative expression levels of cytokines IL-1β (**G**), IL-6 (**H**) and TNF-α (**I**) in the prefrontal cortex, detected by qRT-PCR, respectively. **(J)** The total distance traveled in the open field test by the mice with AAV-mediated AU020206 expression is compared with that of the controls (p = 0.10). **(K)** The time spent in the center zone of the open field test by the mice with AAV-mediated AU020206 expression is compared with that of the controls (p = 0.02). **(L)** The number of marbles buried during the marble burying test by the mice with AAV-mediated AU020206 expression is compared with that of the controls (p = 0.01). **(M)** The open arm residence time in the elevated plus maze test of the mice with AAV-mediated AU020206 expression is compared with that of the controls (p = 0.03). **(N)** The immobility time in the forced swimming test of the mice with AAV-mediated AU020206 expression is compared with that of the controls (p = 0.33). **(O)** The schematic of AU020206-IRFs-STAT1-cytokine axis. It shows that MIA-induced AU020206 binds IRF1/IRF2 to regulate the transcription of STAT1, which further promotes the transcription of cytokines, leading to the anxiety behaviors of the MIA female offspring. For molecular experiments, data are shown as the mean ± SD of three independent experiments (n = 3 for each group); For behavior tests, data are presented as boxplots showing the median, the quantiles, the 5th-95th percentile whiskers (n = 8 for each group). The data values are shown as dots along the boxes. Student's t-test; ns: not significant, * p < 0.05, ** p < 0.01, *** p < 0.001.

We generated the AAV viral particles carrying AU020206 (with IBA1 promoter) and stereotaxically injected the AAVs into the right prefrontal lobe of 8-week female mice (**Figure 5A**). We performed immunofluorescent staining of IBA1 to label microglial cells and fluorescence *in situ* hybridization (FISH) to check the expression of AU020206 (**Figure 5B**) and found that AU020206 showed overexpression in the prefrontal cortex of the mice with the stereotaxic injection of AAV-AU020206 (**Figure 5C**). Then, we performed qRT-PCR on the total RNAs isolated from the prefrontal cortex tissues 21 days after the injection and found that AU020206 overexpression did not influence the expression of IRF1 (**Figure 5D**) and IRF2 (**Figure 5E**), but increased the expression levels of STAT1 (**Figure 5F**) and cytokines IL-1β, IL-6 and TNF-α (**Figure 5G-I**). We further conducted behavioral tests to evaluate the behaviors observed in the MIA female offspring. The mice with AU020206 overexpression exhibited no significant difference in locomotion compared to the controls (n = 8 for each group, p = 0.10, **Figure 5J**) and showed decreased time spent in the center zone of the open field (n = 8 for each group, p = 0.02, **Figure 5K**). The treated mice buried more marbles in the marble burying test (n = 8 for each group, p = 0.01, **Figure 5L**) and spent less time in the open arms of the elevated plus maze test (n = 8 for each group, p = 0.03, **Figure 5M**). These behaviors suggest that the mice with AU020206 overexpression have increased anxiety. We also assessed the depression-like behavior of the mice with AU020206 overexpression using the forced swimming test, and the treated mice did not show a significant difference in the immobility time (n = 8 for each group, p = 0.33, **Figure 5N**). These results have verified that AU020206 contributes to cytokine production through the upregulation of STAT1 in the brain, leading to the observed abnormal behaviors of the MIA female offspring (**Figure 5O**).

## Discussion

The clinical epidemiology investigation demonstrates that MIA induced by prenatal infection of viruses or bacterial pathogens is associated with psychiatric disorders in the later life of the offspring 13, 15-17. Well-recognized differences in psychiatric disorders exist between the male and female 64, 65. Males have a higher prevalence of autism and schizophrenia 66, 67, while females are more prone to anxiety disorder and major depression disorder 68-70. MIA animal models have become practical experimental tools for studying the maldevelopment and dysfunction of the brain subjected to maternal infection during gestation 20-23. MIA may prime the brain at the embryonic stage to establish primary changes that make the brain more vulnerable to a second environmental insult 12. Sex differences in MIA models by different researchers using different protocols 32 suggest that MIA may have different effects on the brains of female and male offspring. In this study, we only observed significantly increased anxiety behaviors and a large number of DEGs in female offspring, we observed no significant behavioral changes and only a small number of DEGs in the male offspring (**Figure 1-2**). These results are consistent with the observation of a higher prevalence of anxiety in women.

To investigate the molecular mechanism underlying the anxiety behaviors in the MIA female offspring, we performed RNA-seq on the whole brain of mice at postnatal 8 weeks. The DEGs of the MIA female offspring are enriched with the candidate genes of anxiety, depression and autism (**Figure 2E-G**). The DEGs are also enriched with immune pathways and immune genes (**Figure S2A, 2M**). Anxiety is comorbid with multiple psychiatric disorders such as depression 71, autism 72, and schizophrenia 73. Anxiety is also associated with increased inflammation 74.

We searched the TFs that may be involved in the brain dysfunction of MIA female offspring and identified a cluster of TFs, including IRF family members and STAT1 (**Figure 3A-B**), which target most DEGs (**Figure S3A**). These TFs form an IRF-STAT1 regulatory network in controlling cytokine production (**Figure 3D-G**). The IRF family and STAT1 are known to play an important role in immune response 75-79 and are associated with anxiety and depression 80-82.

IRF1 and IRF2 are only slightly upregulated in the MIA offspring (**Figure S3B**), which may not fully explain the STAT1's upregulation. Transcriptional activities can be regulated, not only by TFs, but also by other factors, such as lncRNAs, microRNAs, and epigenetic modifications. A recent study found that MIA induces methylome remodeling in the brain of the offspring linking to anxiety-like and depression-like behaviors 83. We identified the DE lncRNA AU020206 that regulates the “IRFs-STAT1-cytokine axis” and further verified the axis in the BV2 cell line (**Figure 4**). The overexpression of AU020206 in the prefrontal cortex increases the expression levels of STAT1 and cytokines (**Figure 5F-I**) and causes anxiety behaviors in the female mice (**Figure 5K-M**). This study establishes the “AU020206-IRFs-STAT1-cytokine axis” involved in the abnormal immunity in the brain of MIA female offspring, which serves as a potential therapeutic target of psychiatric disorders induced by MIA. We did not investigate why the “AU020206-IRF1/2-STAT1-cytokine” axis was only upregulated in the brain of female offspring, not upregulated in the brain of male offspring. The mechanism underlying the sex differences in MIA offspring should be further studied.

## Supplementary Material

Supplementary figures.

Supplementary tables.

## Abbreviations

MIA: maternal immune activation
NDDs: neurodevelopmental disorders
CNS: central nervous system
SPF: specific pathogen-free
LPS: lipopolysaccharide
GD16: gestational day 16
P56: postnatal day 56
EPMT: elevated plus maze test
OFT: open field test
MBT: marble burying test
PPI: prepulse inhibition test
SPT: sucrose preference test
FST: forced swimming test
ASD: autism spectrum disorder
SCZ: schizophrenia
FDR: false discovery rate
PCA: principal component analysis
RRHO: rank-rank hypergeometric overlap
DEGs: differentially expressed genes
TFs: transcription factors
ChIP: chromatin immunoprecipitation assay
ChIRP: chromatin isolation by RNA purification
RIP: RNA immunoprecipitation
FISH: fluorescence *in situ* hybridization

## References

[1] Uher R, Zwicker A (2017). Etiology in psychiatry: embracing the reality of poly-gene-environmental causation of mental illness. World Psychiatry.

[2] Guma E, Bordeleau M, Gonzalez Ibanez F, Picard K, Snook E, Desrosiers-Gregoire G (2022). Differential effects of early or late exposure to prenatal maternal immune activation on mouse embryonic neurodevelopment. Proc Natl Acad Sci U S A.

[3] Pena CJ, Kronman HG, Walker DM, Cates HM, Bagot RC, Purushothaman I (2017). Early life stress confers lifelong stress susceptibility in mice via ventral tegmental area OTX2. Science.

[4] Davis J, Eyre H, Jacka FN, Dodd S, Dean O, McEwen S (2016). A review of vulnerability and risks for schizophrenia: Beyond the two hit hypothesis. Neurosci Biobehav Rev.

[5] Mangino M, Roederer M, Beddall MH, Nestle FO, Spector TD (2017). Innate and adaptive immune traits are differentially affected by genetic and environmental factors. Nat Commun.

[6] Zhang XY, Zhou DF, Zhang PY, Wu GY, Cao LY, Shen YC (2002). Elevated interleukin-2, interleukin-6 and interleukin-8 serum levels in neuroleptic-free schizophrenia: association with psychopathology. Schizophr Res.

[7] Thomas AJ, Davis S, Morris C, Jackson E, Harrison R, O'Brien JT (2005). Increase in interleukin-1beta in late-life depression. Am J Psychiatry.

[8] Al-Ayadhi LY, Mostafa GA (2012). Elevated serum levels of interleukin-17A in children with autism. J Neuroinflammation.

[9] Birnbaum R, Jaffe AE, Chen Q, Shin JH, BrainSeq C, Kleinman JE (2018). Investigating the neuroimmunogenic architecture of schizophrenia. Mol Psychiatry.

[10] Pantazatos SP, Huang YY, Rosoklija GB, Dwork AJ, Arango V, Mann JJ (2017). Whole-transcriptome brain expression and exon-usage profiling in major depression and suicide: evidence for altered glial, endothelial and ATPase activity. Mol Psychiatry.

[11] Gupta S, Ellis SE, Ashar FN, Moes A, Bader JS, Zhan J (2014). Transcriptome analysis reveals dysregulation of innate immune response genes and neuronal activity-dependent genes in autism. Nat Commun.

[12] Gao Y, Li Y, Li S, Liang X, Ren Z, Yang X (2021). Systematic discovery of signaling pathways linking immune activation to schizophrenia. iScience.

[13] Fluegge K (2016). Maternal infection during pregnancy, risk of offspring autism, and the role of bacterial denitrification. Brain Behav Immun.

[14] Han VX, Patel S, Jones HF, Dale RC (2021). Maternal immune activation and neuroinflammation in human neurodevelopmental disorders. Nat Rev Neurol.

[15] Brown AS, Patterson PH (2011). Maternal infection and schizophrenia: implications for prevention. Schizophr Bull.

[16] Parboosing R, Bao Y, Shen L, Schaefer CA, Brown AS (2013). Gestational influenza and bipolar disorder in adult offspring. JAMA Psychiatry.

[17] Murphy SK, Fineberg AM, Maxwell SD, Alloy LB, Zimmermann L, Krigbaum NY (2017). Maternal infection and stress during pregnancy and depressive symptoms in adolescent offspring. Psychiatry Res.

[18] Quagliato LA, de Matos U, Nardi AE (2021). Maternal immune activation generates anxiety in offspring: A translational meta-analysis. Transl Psychiatry.

[19] Brown AS, Begg MD, Gravenstein S, Schaefer CA, Wyatt RJ, Bresnahan M (2004). Serologic evidence of prenatal influenza in the etiology of schizophrenia. Arch Gen Psychiatry.

[20] Depino AM (2015). Early prenatal exposure to LPS results in anxiety- and depression-related behaviors in adulthood. Neuroscience.

[21] Ronovsky M, Berger S, Molz B, Berger A, Pollak DD (2016). Animal Models of Maternal Immune Activation in Depression Research. Curr Neuropharmacol.

[22] Lombardo MV, Moon HM, Su J, Palmer TD, Courchesne E, Pramparo T (2018). Maternal immune activation dysregulation of the fetal brain transcriptome and relevance to the pathophysiology of autism spectrum disorder. Mol Psychiatry.

[23] Knuesel I, Chicha L, Britschgi M, Schobel SA, Bodmer M, Hellings JA (2014). Maternal immune activation and abnormal brain development across CNS disorders. Nat Rev Neurol.

[24] Estes ML, McAllister AK (2016). Maternal immune activation: Implications for neuropsychiatric disorders. Science.

[25] Estes ML, McAllister AK (2016). IMMUNOLOGY. Maternal TH17 cells take a toll on baby's brain. Science.

[26] Haida O, Al Sagheer T, Balbous A, Francheteau M, Matas E, Soria F (2019). Sex-dependent behavioral deficits and neuropathology in a maternal immune activation model of autism. Transl Psychiatry.

[27] Canetta S, Bolkan S, Padilla-Coreano N, Song LJ, Sahn R, Harrison NL (2016). Maternal immune activation leads to selective functional deficits in offspring parvalbumin interneurons. Mol Psychiatry.

[28] Carlezon WA Jr, Kim W, Missig G, Finger BC, Landino SM, Alexander AJ (2019). Maternal and early postnatal immune activation produce sex-specific effects on autism-like behaviors and neuroimmune function in mice. Sci Rep.

[29] Meyer U, Nyffeler M, Engler A, Urwyler A, Schedlowski M, Knuesel I (2006). The time of prenatal immune challenge determines the specificity of inflammation-mediated brain and behavioral pathology. J Neurosci.

[30] Xu DX, Wang H, Zhao L, Ning H, Chen YH, Zhang C (2007). Effects of low-dose lipopolysaccharide (LPS) pretreatment on LPS-induced intra-uterine fetal death and preterm labor. Toxicology.

[31] Hsueh PT, Wang HH, Liu CL, Ni WF, Chen YL, Liu JK (2017). Expression of cerebral serotonin related to anxiety-like behaviors in C57BL/6 offspring induced by repeated subcutaneous prenatal exposure to low-dose lipopolysaccharide. PLoS One.

[32] Xuan IC, Hampson DR (2014). Gender-dependent effects of maternal immune activation on the behavior of mouse offspring. PLoS One.

[33] Smith SE, Li J, Garbett K, Mirnics K, Patterson PH (2007). Maternal immune activation alters fetal brain development through interleukin-6. J Neurosci.

[34] Kalish BT, Kim E, Finander B, Duffy EE, Kim H, Gilman CK (2021). Maternal immune activation in mice disrupts proteostasis in the fetal brain. Nat Neurosci.

[35] Dahlgren J, Samuelsson AM, Jansson T, Holmang A (2006). Interleukin-6 in the maternal circulation reaches the rat fetus in mid-gestation. Pediatr Res.

[36] Hadar R, Dong L, Del-Valle-Anton L, Guneykaya D, Voget M, Edemann-Callesen H (2017). Deep brain stimulation during early adolescence prevents microglial alterations in a model of maternal immune activation. Brain Behav Immun.

[37] Corradini I, Focchi E, Rasile M, Morini R, Desiato G, Tomasoni R (2018). Maternal Immune Activation Delays Excitatory-to-Inhibitory Gamma-Aminobutyric Acid Switch in Offspring. Biol Psychiatry.

[38] Giovanoli S, Weber-Stadlbauer U, Schedlowski M, Meyer U, Engler H (2016). Prenatal immune activation causes hippocampal synaptic deficits in the absence of overt microglia anomalies. Brain Behav Immun.

[39] Colonna M, Butovsky O (2017). Microglia Function in the Central Nervous System During Health and Neurodegeneration. Annu Rev Immunol.

[40] Park GH, Noh H, Shao Z, Ni P, Qin Y, Liu D (2020). Activated microglia cause metabolic disruptions in developmental cortical interneurons that persist in interneurons from individuals with schizophrenia. Nat Neurosci.

[41] Koyama R, Ikegaya Y (2015). Microglia in the pathogenesis of autism spectrum disorders. Neurosci Res.

[42] Watkins CC, Sawa A, Pomper MG (2014). Glia and immune cell signaling in bipolar disorder: insights from neuropharmacology and molecular imaging to clinical application. Transl Psychiatry.

[43] Yirmiya R, Rimmerman N, Reshef R (2015). Depression as a microglial disease. Trends Neurosci.

[44] Bolger AM, Lohse M, Usadel B (2014). Trimmomatic: a flexible trimmer for Illumina sequence data. Bioinformatics.

[45] Dobin A, Davis CA, Schlesinger F, Drenkow J, Zaleski C, Jha S (2013). STAR: ultrafast universal RNA-seq aligner. Bioinformatics.

[46] Anders S, Pyl PT, Huber W (2015). HTSeq-a Python framework to work with high-throughput sequencing data. Bioinformatics.

[47] Robinson MD, McCarthy DJ, Smyth GK (2010). edgeR: a Bioconductor package for differential expression analysis of digital gene expression data. Bioinformatics.

[48] Love MI, Huber W, Anders S (2014). Moderated estimation of fold change and dispersion for RNA-seq data with DESeq2. Genome Biol.

[49] Yu G, Wang LG, Han Y, He QY (2012). clusterProfiler: an R package for comparing biological themes among gene clusters. OMICS.

[50] Heinz S, Benner C, Spann N, Bertolino E, Lin YC, Laslo P (2010). Simple combinations of lineage-determining transcription factors prime cis-regulatory elements required for macrophage and B cell identities. Mol Cell.

[51] Rosenblatt J, Stein J (2014). RRHO: Test overlap using the Rank-Rank Hypergeometric test. R package version.

[52] Basu SN, Kollu R, Banerjee-Basu S (2009). AutDB: a gene reference resource for autism research. Nucleic Acids Res.

[53] Abrahams BS, Arking DE, Campbell DB, Mefford HC, Morrow EM, Weiss LA (2013). SFARI Gene 2.0: a community-driven knowledgebase for the autism spectrum disorders (ASDs). Mol Autism.

[54] Yang C, Li J, Wu Q, Yang X, Huang AY, Zhang J (2018). AutismKB 2.0: a knowledgebase for the genetic evidence of autism spectrum disorder. Database (Oxford).

[55] Breuer K, Foroushani AK, Laird MR, Chen C, Sribnaia A, Lo R (2013). InnateDB: systems biology of innate immunity and beyond-recent updates and continuing curation. Nucleic Acids Res.

[56] Kang J, Tang Q, He J, Li L, Yang N, Yu S (2022). RNAInter v4.0: RNA interactome repository with redefined confidence scoring system and improved accessibility. Nucleic Acids Res.

[57] Walf AA, Frye CA (2007). The use of the elevated plus maze as an assay of anxiety-related behavior in rodents. Nat Protoc.

[58] Seibenhener ML, Wooten MC (2015). Use of the Open Field Maze to measure locomotor and anxiety-like behavior in mice. J Vis Exp.

[59] Deacon RM (2006). Digging and marble burying in mice: simple methods for in vivo identification of biological impacts. Nat Protoc.

[60] Ioannidou C, Marsicano G, Busquets-Garcia A (2018). Assessing Prepulse Inhibition of Startle in Mice. Bio Protoc.

[61] Takahashi H, Hashimoto R, Iwase M, Ishii R, Kamio Y, Takeda M (2011). Prepulse inhibition of startle response: recent advances in human studies of psychiatric disease. Clin Psychopharmacol Neurosci.

[62] Liu MY, Yin CY, Zhu LJ, Zhu XH, Xu C, Luo CX (2018). Sucrose preference test for measurement of stress-induced anhedonia in mice. Nat Protoc.

[63] Luu K, Greenhill CJ, Majoros A, Decker T, Jenkins BJ, Mansell A (2014). STAT1 plays a role in TLR signal transduction and inflammatory responses. Immunol Cell Biol.

[64] Christiansen DM, McCarthy MM, Seeman MV (2022). Editorial: Understanding the influences of sex and gender differences in mental disorders. Front Psychiatry.

[65] Green T, Flash S, Reiss AL (2019). Sex differences in psychiatric disorders: what we can learn from sex chromosome aneuploidies. Neuropsychopharmacology.

[66] Napolitano A, Schiavi S, La Rosa P, Rossi-Espagnet MC, Petrillo S, Bottino F (2022). Sex Differences in Autism Spectrum Disorder: Diagnostic, Neurobiological, and Behavioral Features. Front Psychiatry.

[67] Fernando P, Sommer IEC, Hasan A (2020). Do we need sex-oriented clinical practice guidelines for the treatment of schizophrenia?. Curr Opin Psychiatry.

[68] McLean CP, Asnaani A, Litz BT, Hofmann SG (2011). Gender differences in anxiety disorders: prevalence, course of illness, comorbidity and burden of illness. J Psychiatr Res.

[69] Nolen-Hoeksema S (1987). Sex differences in unipolar depression: evidence and theory. Psychol Bull.

[70] Patten SB (2009). Accumulation of major depressive episodes over time in a prospective study indicates that retrospectively assessed lifetime prevalence estimates are too low. BMC Psychiatry.

[71] Fisher JE, Zhou J, Liu AG, Fullerton CS, Ursano RJ, Cozza SJ (2020). Effect of comorbid anxiety and depression in complicated grief on perceived cognitive failures. Depress Anxiety.

[72] White SW, Oswald D, Ollendick T, Scahill L (2009). Anxiety in children and adolescents with autism spectrum disorders. Clin Psychol Rev.

[73] Braga RJ, Reynolds GP, Siris SG (2013). Anxiety comorbidity in schizophrenia. Psychiatry Res.

[74] Felger JC (2018). Imaging the Role of Inflammation in Mood and Anxiety-related Disorders. Curr Neuropharmacol.

[75] Ikushima H, Negishi H, Taniguchi T (2013). The IRF family transcription factors at the interface of innate and adaptive immune responses. Cold Spring Harb Symp Quant Biol.

[76] Honda K, Yanai H, Negishi H, Asagiri M, Sato M, Mizutani T (2005). IRF-7 is the master regulator of type-I interferon-dependent immune responses. Nature.

[77] Savitsky D, Tamura T, Yanai H, Taniguchi T (2010). Regulation of immunity and oncogenesis by the IRF transcription factor family. Cancer Immunol Immunother.

[78] Dai X, Sayama K, Yamasaki K, Tohyama M, Shirakata Y, Hanakawa Y (2006). SOCS1-negative feedback of STAT1 activation is a key pathway in the dsRNA-induced innate immune response of human keratinocytes. J Invest Dermatol.

[79] Karst SM, Wobus CE, Lay M, Davidson J, Virgin HWt (2003). STAT1-dependent innate immunity to a Norwalk-like virus. Science.

[80] Zheng M, Li K, Chen T, Liu S, He L (2021). Geniposide protects depression through BTK/JAK2/STAT1 signaling pathway in lipopolysaccharide-induced depressive mice. Brain Res Bull.

[81] Chiang JJ, Cole SW, Bower JE, Irwin MR, Taylor SE, Arevalo J (2019). Depressive symptoms and immune transcriptional profiles in late adolescents. Brain Behav Immun.

[82] Li J, Pang Y, Du Y, Xia L, Chen M, Fan Y (2023). Lack of interferon regulatory factor 3 leads to anxiety/depression-like behaviors through disrupting the balance of neuronal excitation and inhibition in mice. Genes Dis.

[83] Ma L, Wang F, Li Y, Wang J, Chang Q, Du Y (2023). Brain methylome remodeling selectively regulates neuronal activity genes linking to emotional behaviors in mice exposed to maternal immune activation. Nat Commun.

