# Supplementary material for: Maternal immune activation upregulates the AU020206-IRFs-STAT1 axis in modulating cytokine production in the brain
Source: Theranostics. 2024 Sep 3;14(14):5682–97. doi: 10.7150/thno.96110 (PMC11413792; doi:10.7150/thno.96110)
Supplement: Supplementary file 1 — Supplementary figures. [file thnov14p5682s1.pdf]

Supplemental information

Supplemental tables

Table S1 Expressed genes read count

Table S2 Differentially expressed gene list of female offspring

Table S3 Differentially expressed gene list of male offspring

Table S4 Psychiatric disorder genes

Table S5 Immune genes

Table S6 Identified motifs of TFs

Table S7 TF-Target network

Table S8 Primer and siRNA sequences

Supplemental figures

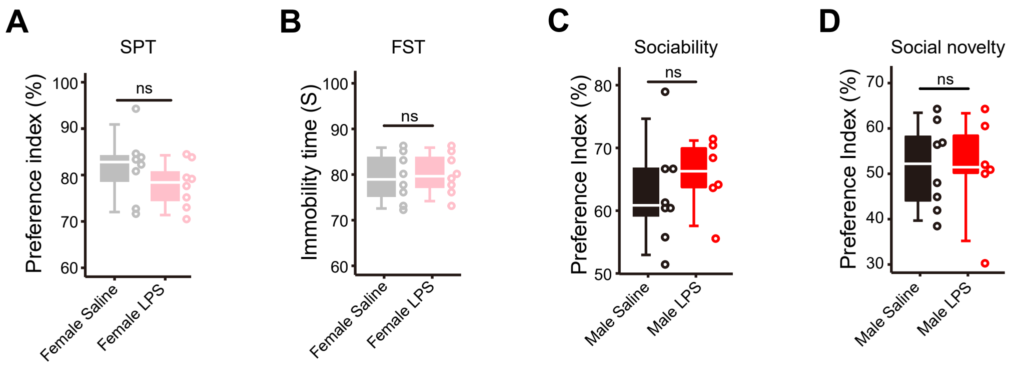

Figure S1 Maternal immune activation increases anxiety-like behaviors in female offspring, but not in the male offspring (See also Figure. 1)

(A) The sucrose preference test (SPT) of the female offspring. The sucrose consumed by the MIA female offspring is compared with that of the controls (p = 0.24, n = 8 for each group).

(B) The forced swimming test (FST) of the female offspring. The immobility time of the female MIA offspring is compared with that of the controls (p = 0.73, n = 8 for each group).

(C) The three-chamber test of the male offspring. The social preference index of MIA male offspring is compared with that of the controls in the 10-minute sociability phase (p = 0.48, n = 8 for the saline group, n = 6 for the LPS group). The Sniffing time is plotted as a social preference index: timeS1/(timeS1+timeO).

(D) The three-chamber test of the male offspring. The social preference index of MIA male offspring is compared with that of the controls in the 10-min social novelty phase (p = 0.96, n = 8 for the saline group, n = 6 for the LPS group). The Sniffing time is plotted as a social preference index: time S2/(time S1+time S2).

Data are presented as boxplots showing the median, the quantiles, and the 5th-95th percentile whiskers. The data values are shown as dots along the boxes, student's t-test, ns: not significant, $p > 0.05$.

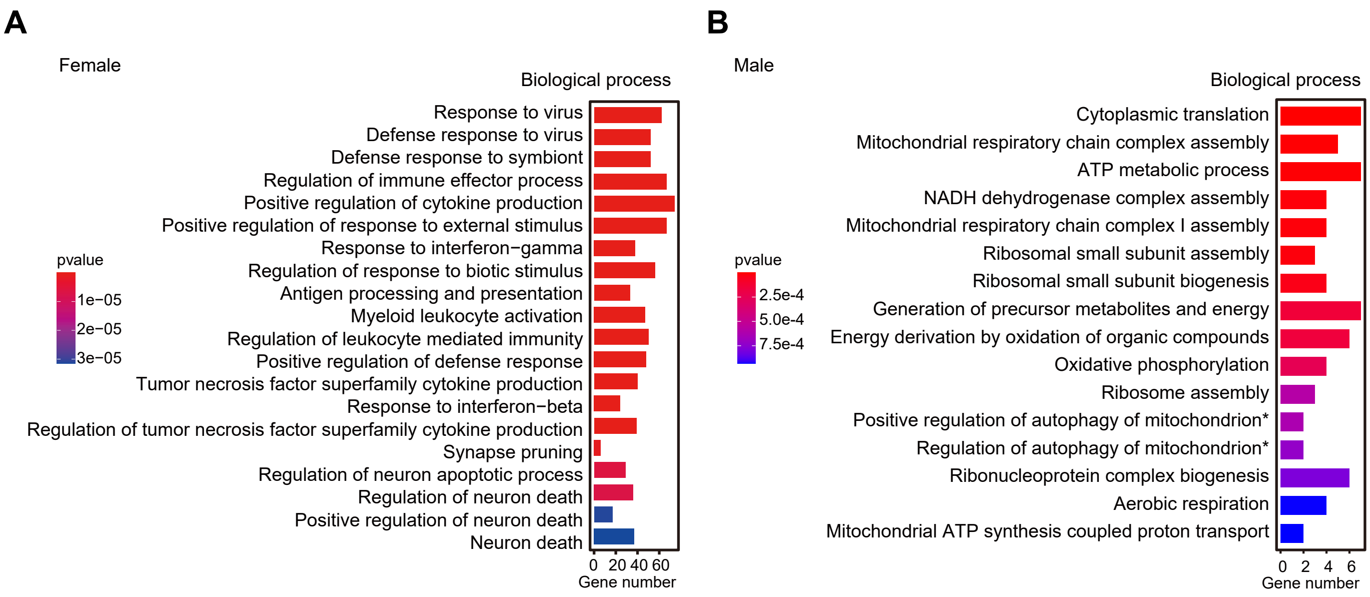

Positive regulation of autophagy of mitochondrion*:
Positive regulation of autophagy of mitochondrion in response to mitochondrial depolarization
Regulation of autophagy of mitochondrion*:
Regulation of autophagy of mitochondrion in response to mitochondrial depolarization

Figure S2 The transcriptomic dysregulation in the brain of the MIA offspring (See also Figure. 2)

(A) The enrichment of GO biological processes with the DEGs of the MIA female offspring. The colors represent the p-value.

(B) The enrichment of GO biological processes with the DEGs of the MIA male offspring. The colors represent the p-value.

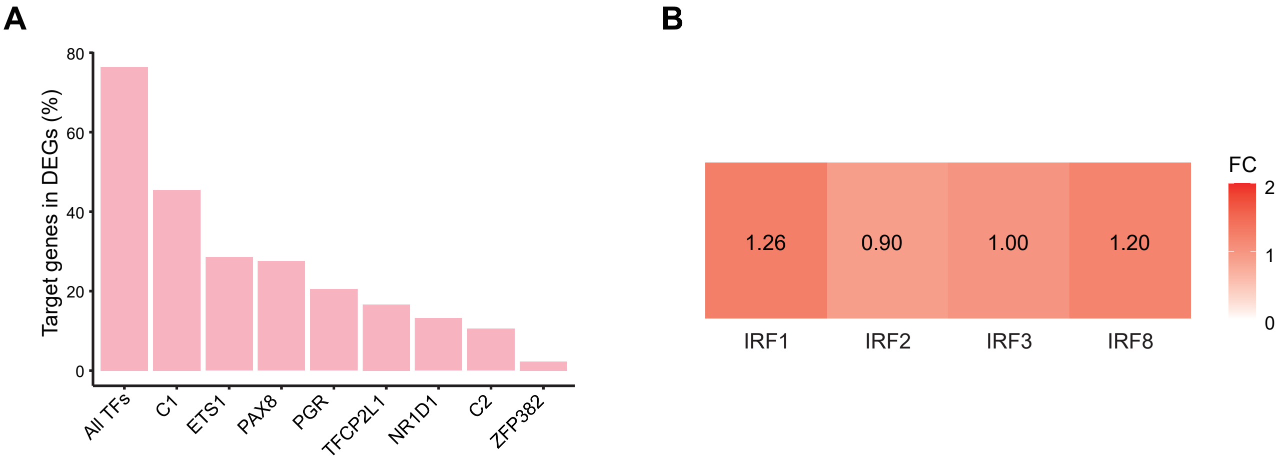
A
Target genes in DEGs (%)
80
60
40
20
0
All TFs
C1
ETS1
PAX8
PGR
TFCP2L1
NR1D1
C2
ZFP382
B
1.26
0.90
1.00
1.20
IRF1
IRF2
IRF3
IRF8
FC
2
1
0

Figure S3 The IRFs-STAT1 regulatory network controls the expression of genes in the brain of MIA female offspring (See also Figure 3)

(A) The proportion of target genes for different clusters TFs in 1,094 DEGs of MIA female offspring.

(B) The heat map of IRF1, IRF2, IRF3 and IRF8 which regulate STAT1. The Color shows the fold change of TFs.

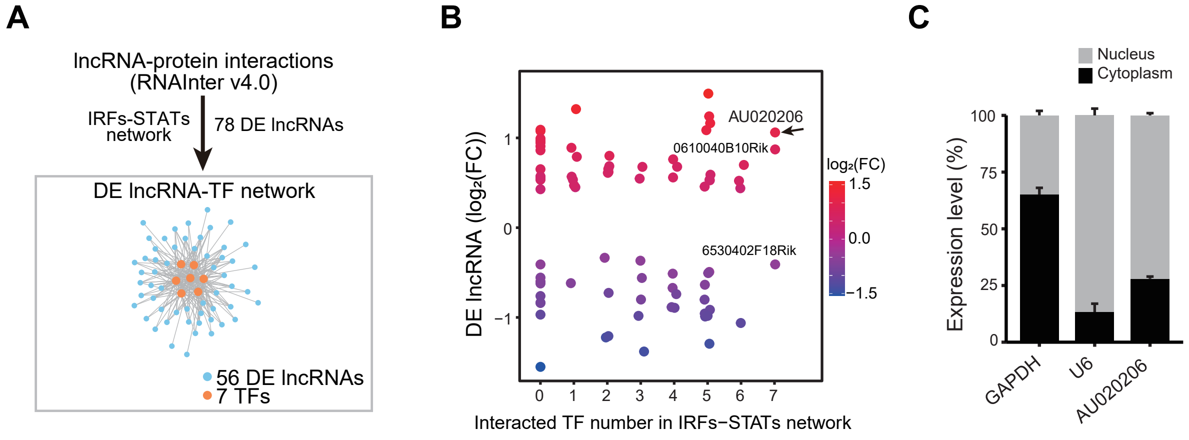

A
lncRNA-protein interactions
(RNAInter v4.0)
IRFs-STATs network
78 DE lncRNAs
DE lncRNA-TF network
56 DE lncRNAs
7 TFs
B
AU020206
0610040B10Rik
6530402F18Rik
log₂(FC)
1.5
0.0
−1.5
DE lncRNA (log₂(FC))
Interacted TF number in IRFs−STATs network
C
Nucleus
Cytoplasm
Expression level (%)
GAPDH
U6
AU020206

Figure S4 AU020206 interacts with IRF1/IRF2 to regulate the transcription of STAT1 in controlling cytokine production (see also Figure 4)

(A) The construction of DE lncRNA-TF network. The IRFs-STATs network and 78 differentially expressed lncRNAs in the DEGs are mapped into the published lncRNA-protein interaction (RNAInter v4.0 [1]) to retrieve the DE lncRNA-TF network. Orange nodes are TFs, and blue nodes are DE lncRNAs.

(B) The number of interacted TF and differential expression levels for 56 DE lncRNAs in the DE lncRNA-TF network. The x-axis is the number of interacted TF in the IRFs-STATs network, the y-axis is the log2(fold change) of DE lncRNAs. The node colors are log2(fold change) of DE lncRNAs.

(C) The subcellular localization of AU020206 in BV2 cells detected by cell fractionation assays. U6: nucleus marker; GAPDH: cytoplasm marker.

Reference

1. Kang J, Tang Q, He J, Li L, Yang N, Yu S, et al. RNAInter v4.0: RNA interactome repository with redefined confidence scoring system and improved accessibility. Nucleic Acids Res. 2022; 50: D326-D32.
